# A Novel Approach for Detecting Rotational Angles of a Precision Spherical Joint Based on a Capacitive Sensor

**DOI:** 10.3390/mi10050280

**Published:** 2019-04-26

**Authors:** Wen Wang, He Yang, Min Zhang, Zhanfeng Chen, Guang Shi, Keqing Lu, Kui Xiang, Bingfeng Ju

**Affiliations:** 1 School of Mechanical Engineering, Hangzhou Dianzi University, Hangzhou 310018, China; wangwn@hdu.edu.cn (W.W.); zhangmme@foxmail.com (M.Z.); czf@hdu.edu.cn (Z.C.); shiguang@hdu.edu.cn (G.S.); lkq@hdu.edu.cn (K.L.); 2 School of Mechanical Engineering, Zhejiang University, Hangzhou 310027, China; snowboy2001102@163.com (K.X.); mbfju@zju.edu.cn (B.J.); 3 State Key Lab of Fluid Power & Mechatronic Systems, Zhejiang University, Hangzhou 310027, China

**Keywords:** capacitive sensor, spherical joint, rotational angle, angular motion, orientation detection

## Abstract

Precision spherical joints are commonly employed as multiple degree-of-freedom (DOF) mechanical hinges in many engineering applications, e.g., robots and parallel manipulators. Real-time and precise measurement of the rotational angles of spherical joints is not only beneficial to the real-time and closed-loop control of mechanical transmission systems, but also is of great significance in the prediction and compensation of their motion errors. This work presents a novel approach for rotational angle measurement of spherical joints with a capacitive sensor. First, the 3-DOF angular motions of a spherical joint were analyzed. Then, the structure of the proposed capacitive sensor was presented, and the mathematical model for the rotational angles of a spherical joint and the capacitance of the capacitors was deduced. Finally, the capacitance values of the capacitors at different rotations were simulated using Ansoft Maxwell software. The simulation results show that the variation in the simulated capacitance values of the capacitors is similar to that of the theoretical values, suggesting the feasibility and effectiveness of the proposed capacitive detection method for rotational angles of spherical joints.

## 1. Introduction

As three-degrees-of-freedom (3-DOF) mechanical hinges with compact and flexible structures, precision spherical joints are widely employed in many engineering applications, such as industrial robots and parallel manipulators [1,2,3]. In the robotic manipulators, the employment of 3-DOF spherical joints, instead of the single DOF joints, could reduce the joint number and improve the transmission accuracy and the motion flexibility. With the rapid development of robotics, an emerging demand is to achieve the real-time and closed-loop control of multiple DOF motion systems [4,5,6]. This requires a highly accurate and real-time measurement of the rotational angles of precision spherical joints. 

Although studies on the rotational angle detection of spherical joints have been rarely reported, many methods have been proposed for the orientation measurement of a spherical motor. Lee and Pei [7] proposed a 3-DOF measurement system for the orientation detection of a spherical rotor. The system consists of a sliding block, two sliding guides, and three single-axis encoders. Each rotary encoder is used to detect individual single-DOF rotation. Although capable of achieving high-precision measurements, three encoders could enlarge the mechanical structure and introduce undesirable friction and additional inertia. To eliminate this drawback, noncontact methodologies have been proposed for the orientation measurement. One approach is to use vision-based orientation measurement system [8]. The spherical motion can be detected by using an image-processing system to capture special grid patterns on the spherical body. However, the challenge encountered is the requirement of spherical surface gridding with high resolution. Another method is to adopt optical sensors [9,10]. The relative motion of the surface is determined by analyzing the differences in two consecutive captured images. The drawback of this method is that the output signal of optical sensor is very sensitive to the clearance and relative motion between the rotor and the sensor. A substitute for the light-based system mentioned above is Hall-effect sensors [11,12,13]. The angular motion can be calculated by measuring the magnetic flux density at certain points around the rotor. However, the small variation in the magnetic field with the rotation of the rotor leads to a low measurement resolution.

Compared to other detection methods, a capacitive sensor has the advantages of high accuracy, good dynamic performance, relatively low cost, and simplicity. Ahn et al. [14,15] developed a cylindrical capacitive sensor to detect the radial and axial motion of rotating machinery. The displacements could be obtained by calculating the variation in eight capacitors. Han et al. [16] conducted a capacitive detection of the radial movement of a spherical rotor. Six pairs of electrodes are distributed symmetrically around the spherical rotor. The spatial displacements of the rotor are achieved by measuring the change in the spherical capacitance. Anandan and George [17] proposed a capacitive sensor to measure the linear and angular displacements of a rotational shaft. The linear displacement and rotation are obtained by detecting the capacitance variation of four semi-hollow, cylindrical capacitors. Although many capacitive sensors have been proposed for motion detection, the capacitive detection of rotational angles of precision spherical joints has been rarely reported.

Thus, this work proposes a novel detection method for the rotational angles of a spherical joint based on a capacitive sensor. First, 3-DOF angular motions of a spherical joint are analyzed in Section 2. Then, Section 3 introduces the structure and mathematical model of the capacitive sensor. Further, the capacitance of the capacitive sensor is simulated with Ansoft Maxwell 16.0 in Section 4 & Section 5. Finally, the main conclusions are presented in Section 6.

## 2. 3-DOF Angular Motions of a Spherical Joint 

**Figure 1:**
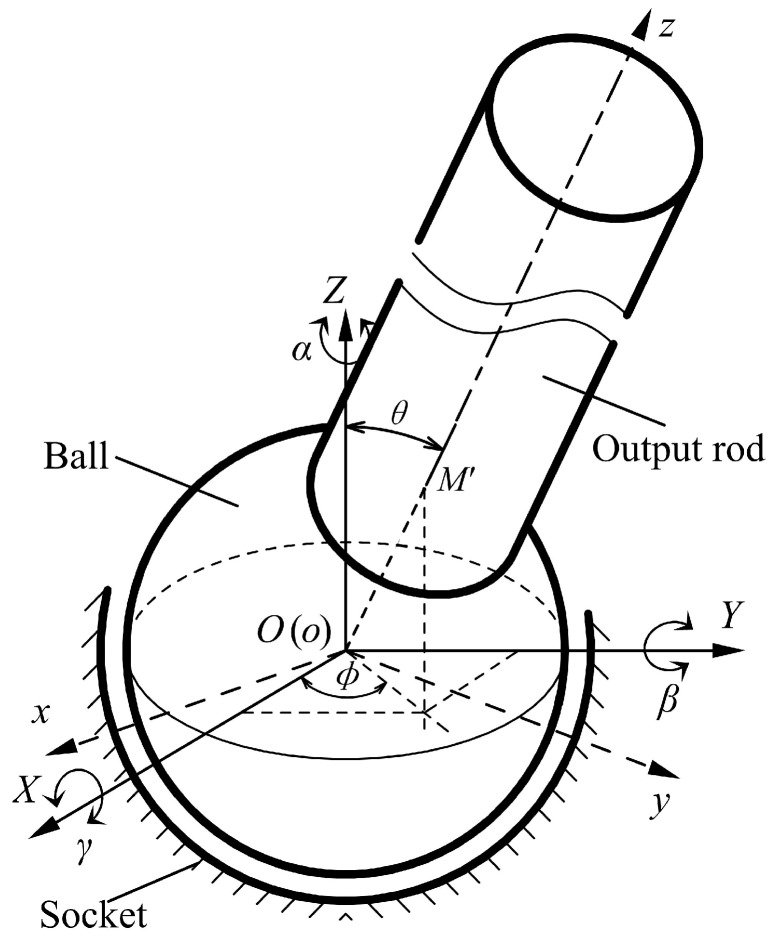
Schematic model for the three-degrees-of-freedom (3-DOF) angular motions of a spherical joint.

To explore the angular motions of a spherical joint, we define two coordinate systems, i.e., the moving coordinate system *oxyz* and the fixed coordinate system *OXYZ* (Figure 1). The former is defined as the ball with the origin *o* at the center of the ball, while the latter is defined as the socket with the origin *O* at the spherical center of the socket. Assuming that the ball has no eccentric displacement in the socket, two original points *O* and *o* superpose with each other. The output rod of the spherical joint has a 3-DOF rotation about the center of the socket, and the moving coordinate system coincides with the fixed coordinate system at the initial position. Thus, the spatial rotation of the spherical joint can be described by the relative position of the fixed and moving coordinate systems.

According to the description methods of robot kinematics, the 3-DOF angular motions of a spherical joint can be expressed by the *RPY* angles. Initially, the moving coordinate system *oxyz* coincides with the fixed coordinate system *OXYZ*. As the spherical joint rotates, the coordinate system *oxyz* firstly rotates by an angle *γ* about the *X*-axis, and it subsequently rotates by an angle *β* about the *Y*-axis, and further rotates by an angle *α* about the *Z*-axis. As a result, the coordinate system *oxyz* arrives at the final position, which can be defined as (*γ*, *β*, *α*) as a type of *RPY* angle. In addition, the 3-DOF rotation of the spherical joint can be described by the rotations about three directions, i.e., rotation about the *X*-axis, rotation about the *Y*-axis, and rotation about the *Z*-axis. In addition, the output rod of the spherical joint can rotate by 360° about the rod centerline (*Z*-axis), and this angle can be accurately measured by the rotary encoder installed on the output rod. As such, the main challenge of the spatial rotation measurement of the spherical joint is to detect 2-DOF angular motions, i.e., rotational angle about the *X*-axis (*γ*) and that about the *Y*-axis (*β*).

Initially, the intersection point of the ball surface and the centerline of the output rod is defined as *M* (0, 0, *r*), where *r* is the radius of the ball. As the spherical joint rotates by an *RPY* angle (*γ*, *β*, 0), the point *M* moves to the point *M’* (*x*, *y*, *z*). According to the coordinate transformations, the coordinates of *M’* can be given as follows:

(1) $$\left\{ \begin{array}{l} x=r\sin\beta \cos\gamma \\ y=-r\sin\gamma \\ z=r\cos\beta \cos\gamma \end{array}\right.$$

Since the rotation of the output rod about the *Z*-axis has no effect on the coordinate of the point *M’* (*x*, *y*, *z*), it can be expressed as the following equations in terms of inclination angle (*θ*), azimuthal angle (*φ*), and the radius of the ball (*r*) in the spherical coordinate system,

(2) $$\left\{ \begin{array}{l} x=r\sin\theta \cos\varphi \\ y=r\sin\theta \sin\varphi \\ z=r\cos\theta \end{array}\right.$$

By using Equations (1) and (2), the relation between the *RPY* angles (*γ* and *β*) and the rotational angles in the spherical coordinate system (*θ* and *φ*) can be derived as follows:

(3) $$\left\{ \begin{array}{l} \theta =\arccos(\cos\beta \cos\gamma )\\ \varphi =-\mathrm{arccot}(\sin\beta \cot\gamma ) \end{array}\right.$$

Therefore, the rotational angle detection of a spherical joint can be simplified to the measurement of the rotational angles about the *X-* and *Y*-axes (*γ* and *β*). In addition, the rotation of the spherical joint can be also described using the inclination angle (*θ*) and azimuthal angle (*φ*) in the spherical coordinate system, which can be calculated using the *RPY* angles (*γ* and *β*). 

## 3. Detection Method

### 3.1. Measuring Principle

**Figure 2:**
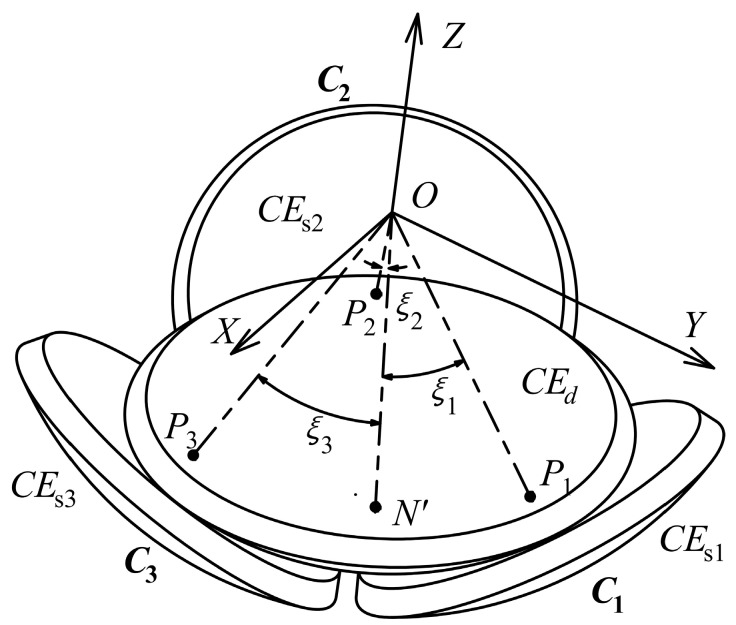
Structural model of the capacitive sensor and corresponding coordinate system.

An area-varying capacitive sensor with a spherically coronal structure is proposed to detect the 2-DOF angular motions of a spherical joint, i.e., the rotations of the output rod about the *X-* and *Y*-axes, respectively. As shown in Figure 2, the proposed capacitive sensor mainly includes four spherically coronal electrodes, i.e., an excitation electrode (*CE*_d_) and three sensing electrodes (*CE*_s*i*_, *i* = 1, 2, 3). The excitation electrode is applied as a common electrode, which is concentrically attached to the lower surface of the ball of the spherical joint. The point *N* denotes the intersection point of the central axis of the output rod and the outer surface of the excitation electrode *CE*_d_, i.e., the center of the excitation electrode plate. Three sensing electrodes are concentrically fixed on the inner surface of the socket. Each sensing electrode (*CE*_s*i*_, *i* = 1, 2, 3) and the excitation electrode *CE*_d_ form a capacitor (*C_i_*, *i* = 1, 2, 3). The central point of the inner surface of *CE*_s1_, *CE*_s2_, and *CE*_s3_ is defined as *P*_1_, *P*_2_, and *P*_3_, respectively. The main advantage of the spherically coronal electrodes is that it could avoid the effect of the rotation about the rod centerline on the detection of other 2-DOF angular motions. This is because the overlapping area of the excitation electrode plate and each sensing electrode plate remains unchanged as the output rod rotates around its own axis (*z*-axis). Teflon-laced epoxy with self-lubricating and abrasion-resistant properties is used as a dielectric material, which is uniformly deposited on the surfaces of the spherically coronal electrodes. This would produce the insulation between the sensing electrodes and the excitation electrode, and improves the sensitivity of the sensor.

Initially, the output rod of the spherical joint is placed along the vertical direction, that is, the central axis of the output rod is aligned with the axis of the socket. Three capacitors *C*_1_, *C*_2_ and *C*_3_ have the same capacitance value. Once the output rod rotates about the *X-* and *Y*-axes, the effective overlapping area of the excitation electrode plate and each sensing electrode plate is varied, changing the capacitance values of each capacitor. Thus, the angles (*ξ*_1_, *ξ*_2_ or *ξ*_3_) between the centers of three sensing electrode plates and that of the excitation electrode plate could be calculated from the measured capacitance values of the capacitors *C*_1_, *C*_2_, and *C*_3_; then, the rotational angles (*γ* and *β*) of the output rod of the spherical joint about the *X-* and *Y*-axes could be obtained.

For the convenience of presenting the relation between three central angles and two rotational angles, it is assumed that three sensing electrode plates are uniformly distributed along the same latitudinal direction. Thus, the initial angle (*ρ*_0_) between the center of each sensing electrode plate and that of the excitation electrode plate is equal to each other. As shown in Figure 2, at the initial position, the coordinates of the point *N* are (0, 0, -*r*). As the spherical joint rotates by an *RPY* angle (*γ*, *β*, 0), the point *N* moves to point *N’*. Consequently, the angles *ξ*_1_, *ξ*_2_, and *ξ*_3_ are subtended by the arc *N’ P*_1_, *N’ P*_2_, and *N’ P*_3_, respectively. They can be given by the following equations:

(4) $$\begin{array}{ll} \cos\xi_{1}= & 1-\frac{1}{2}[{\left(\sin\beta \cos\gamma \right)}^{2}+\ldots \\ & {\left(\sin\rho_{0}-\sin\gamma \right)}^{2}+{\left(\cos\beta \cos\gamma -\cos\rho_{0}\right)}^{2}] \end{array}$$

(5) $$\begin{array}{ll} \cos\xi_{2}= & 1-\frac{1}{2}[{\left(\sin\beta \cos\gamma -\sin\rho_{0}\cos\frac{\pi }{6}\right)}^{2}+\ldots \\ & {\left(\sin\rho_{0}\sin\frac{\pi }{6}+\sin\gamma \right)}^{2}+{\left(\cos\beta \cos\gamma -\cos\rho_{0}\right)}^{2}] \end{array}$$

(6) $$\begin{array}{ll} \cos\xi_{3}= & 1-\frac{1}{2}[{\left(\sin\beta \cos\gamma +\sin\rho_{0}\cos\frac{\pi }{6}\right)}^{2}+\ldots \\ & {\left(\sin\rho_{0}\sin\frac{\pi }{6}+\sin\gamma \right)}^{2}+{\left(\cos\beta \cos\gamma -\cos\rho_{0}\right)}^{2}] \end{array}$$

Using Equations (5) and (6), we obtain the following:

(7) $$\sin\beta \cos\gamma =\frac{1}{2}\frac{\cos\xi_{2}-\cos\xi_{3}}{\sin\rho_{0}\cos\frac{\pi }{6}}$$

Using Equations (4), (5), and (7), we can derive the equation of the rotation angle *γ*:

(8) $$\sin\gamma =\frac{2\cos\xi_{1}-\cos\xi_{2}-\cos\xi_{3}}{2\sin\rho_{0}(1+\sin\frac{\pi }{6})}$$

By substituting Equation (8) into Equation (7), we obtain the equation of the rotation angle *β*:

(9) $$\sin\beta =\frac{1}{2}\frac{\cos\xi_{2}-\cos\xi_{3}}{\cos\gamma \sin\rho_{0}\cos\frac{\pi }{6}}$$

Thus, according to Equations (8) and (9), the 2-DOF rotation angles (*γ* and *β*) of the spherical joint could be obtained, provided that the angles (*ξ*_1_, *ξ*_2_, or *ξ*_3_) could be calculated from the measured capacitance values of the capacitors *C*_1_, *C*_2_, and *C*_3_.

### 3.2. Mathematical Model of the Capacitive Sensor

To simplify the calculations of the capacitance values, we make the following assumptions: (1) the area element *dS* of spherically coronal plates is assumed as a parallel-plate capacitor with an equal separation distance *d*; (2) it is assumed that the gap *d* is much smaller than the dimensions of the plates; (3) the fringing effect of the capacitive plates is small enough to be ignored. 

By integrating the capacitance formula for the plate capacitors, we can obtain the capacitance value of the spherical capacitors as follows:

(10) $$C_{i}=\frac{\varepsilon }{d}\;\iint_{S_{i}}dS\;(i=1,2,3)$$

where *ε* is the permittivity of the dielectric material between the excitation electrode and the sensing electrode, while *S_i_* denotes the effective overlapping area of the sensing electrodes (*CE*_s*i*_, *i* = 1, 2, 3) and the excitation electrode. 

**Figure 3:**
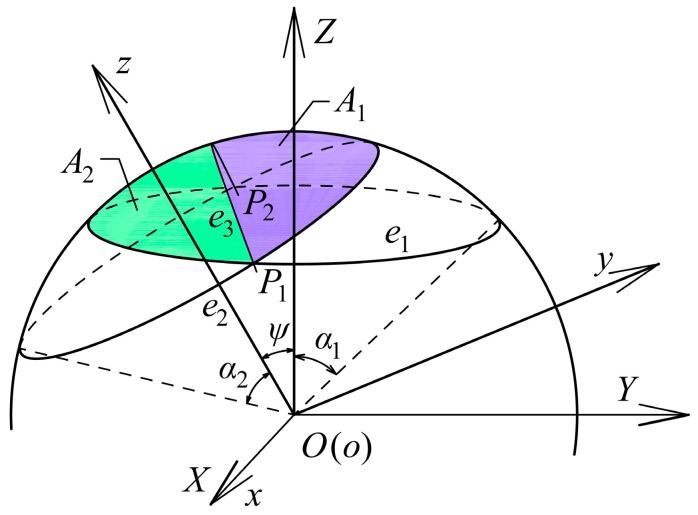
Mathematical model for the overlapping area of the spherically coronal plates.

To approximately calculate the effective overlapping area of the sensing electrodes and the excitation electrode, we assume that the radius of the spherical capacitive plates is equal to that of the ball of the spherical joint (*r*). Figure 3 presents the mathematical model of the overlapping area of two spherically coronal capacitive plates, along with a fixed coordinate system *OXYZ* and a moving coordinate system *oxyz*. The spherical sensing electrode is fixed at the coordinate system *OXYZ* with its central axis along the *Z*-axis and the corresponding sphere center at the origin *O*. The spherical excitation electrode is attached on the coordinate system *oxyz* with its central axis along the *Z*-axis and the corresponding sphere center at the origin *o*. Two origin points *O* and *o* superpose each other. The curves *e*_1_ and *e*_2_ are the boundary of the sensing electrode and the excitation electrode, respectively. The plane *OP*_1_*P*_2_, crossing through the origin *O* and intersection points *P*_1_ and *P*_2_, separates the overlapping area of the two spherical electrodes into two parts, i.e., *A*_1_ and *A*_2_. The curve *e*_3_ is the intersection line of the plane *OP*_1_*P*_2_ and the sphere. Initially, the two coordinate systems superpose each other. If the coordinate system *oxyz* rotates about the *X*-axis with an angle of *ψ*, then the angle between the centers of two spherical capacitive plates is *ψ*. *α*_1_ and *α*_2_ are the central angles of the sensing electrode and the excitation electrode, respectively.

The spherical area *A_i_* (*i =* 1, 2) surrounded by the curve *e_i_* (*i =* 1, 2) and the curve *e*_3_ can be calculated as follows:

(11) $$\begin{array}{l} A_{i}=r^{2}\{\left[1+\mathrm{sgn}(\cos\alpha_{i}\cos\psi -\cos\alpha_{i+1})\right](1-\cos\alpha_{i})\pi +\\ \;2\mathrm{sgn}(\cos\alpha_{i}\cos\psi -\cos\alpha_{i+1})\left[\gamma_{i}\cos\alpha_{i}-\sin^{-1}(\cos\eta_{i}\sin\gamma_{i})\right]\} \end{array}$$

where $\alpha_{3}=\alpha_{1}$, $\eta_{i}=\tan^{-1}\left[\left(\cos\alpha_{i+1}-\cos\alpha_{i}\cos\psi \right)/(\cos\alpha_{i}\sin\psi )\right]$, $\mathrm{sgn}(x)=\left\{ \begin{array}{l} 1\;x>0\\ -1\;x<0 \end{array}\right.$, $\gamma_{i}=\tan^{-1}\left[\frac{\sqrt{{\left(\sin\alpha_{i}\sin\psi \right)}^{2}-{\left(\cos\alpha_{i}\cos\psi -\cos\alpha_{i+1}\right)}^{2}}}{\left|\cos\alpha_{i}\cos\psi -\cos\alpha_{i+1}\right|}\right]$.

Then, the effective overlapping area of two spherical electrode plates can be obtained as follows:

(12) $$S=\sum_{i=1}^{2}A_{i}$$

According to Equations (11) and (12), the effective overlapping area of the excitation electrode and the sensing electrode has a relation with the angle *ψ*, which can be defined as follows:

(13) $$S=r^{2}g(\psi )$$

Considering the special structure of the spherical joints, the boundary of the excitation electrode should not move beyond equatorial plane of the socket when the excitation electrode rotates by the maximal angles. Thus, the central angle *α*_2_ of the excitation electrode should satisfy the condition of *α*_2_ ≤ 45°. On the other hand, the large overlapping area of the capacitive plates could improve the sensitivity of the sensor. Hence, we have *α*_2_ = 45°. To avoid the structure interference of three sensing electrodes, we choose *α*_1_ = 30°, and *ρ*_0_ = 37°. As *ψ* varies from 27° to 47°, *g*(*ψ*) can be fitted into linear function *g’*(*ψ*) with the maximum relative error of 0.467%. The fitting function is given as follows:

*g’*(*ψ*) = −0.9902*ψ* + 1.1506 (14)

Then, by using Equations (10)–(14), the capacitance value of the capacitor can be expressed by:

(15) $$C=\frac{\varepsilon r^{2}}{d}(-0.9902\psi +1.1506)$$

Thus, the relation between the capacitance value of each capacitor and the angle *ψ* between the centers of two capacitive plates is established. In other words, the angles (*ξ*_1_, *ξ*_2_ or *ξ*_3_) between the centers of three sensing electrode plate and that of the excitation electrode plate could be calculated from the measured capacitance values of the capacitors *C*_1_, *C*_2_ and *C*_3_.

## 4. Simulation Setup

**Figure 4:**
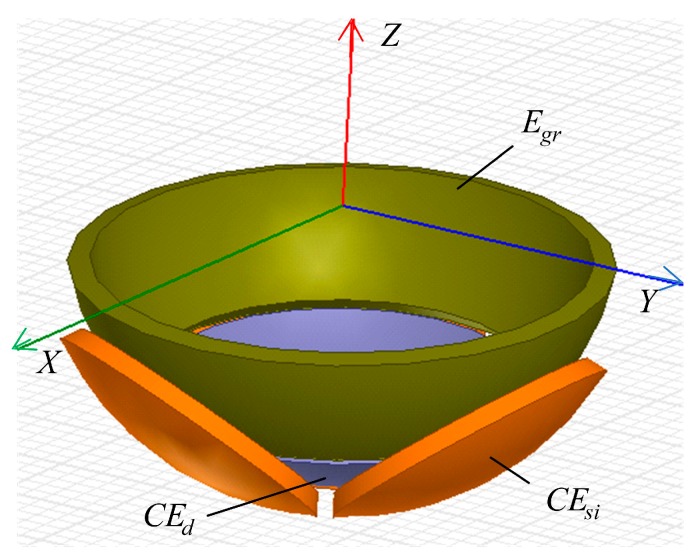
Simulation model of the capacitive sensor with guard ring.

To validate the feasibility of the proposed method, the proposed capacitive sensor was simulated using Ansoft Maxwell. In the practical applications of capacitive sensors, the electric-field lines bend at the edge of the capacitive plates, resulting in the additional capacitance [18]. This phenomenon is referred to as the fringe effect, which could reduce the sensitivity of the sensor and produce nonlinear errors [19]. Thus, the guard ring (*E_gr_*) was set around the spherical capacitive electrodes to reduce the fringe effect on the output of the capacitive sensor. Figure 4 presents the simulation model of the capacitive sensor. It consists of an excitation electrode, a guard ring, and three sensing electrodes. The air was used for the dielectric material between the capacitive plates to simplify the simulation model. The structural parameters of the capacitive sensor are shown in Table 1. It should be pointed out that the electromagnetic shield should be considered in the practical applications. Specifically, in order to reduce the external interference from other metal structures and the environmental noise, the sensing electrodes are shielded by the grounded shell and guard ring, and a dielectric material is placed between the excitation electrode and the ball of spherical joints.

The simulated and theoretical capacitance values of the capacitors *C*_1_, *C*_2_ and *C*_3_ were compared in detail to validate the feasibility of the proposed method. In the simulation, we assume that the spherical excitation electrode (*CE_d_*) firstly rotates by an angle *γ* about the *X*-axis, and then rotates by an angle *β* about the *Y*-axis. The capacitance values of three capacitors with and without the guard ring *E_g_*_r_ are calculated by Ansoft Maxwell. For the theoretical calculation, the angles (*ξ*_1_, *ξ*_2_ and *ξ*_3_) of the capacitive plates can be obtained by substituting the simulation values of *β* and *γ* into Equations (4)–(6). Then, the capacitance value of three capacitors can be calculated according to Equations (8)–(12). Four cases are examined in this work; that is, the output rod of the spherical joint rotates about the *X*-axis before rotating about the *Y*-axis, and the corresponding rotational angles (*γ* and *β*) are shown in Table 2.

## 5. Results and Discussions

**Figure 5:**
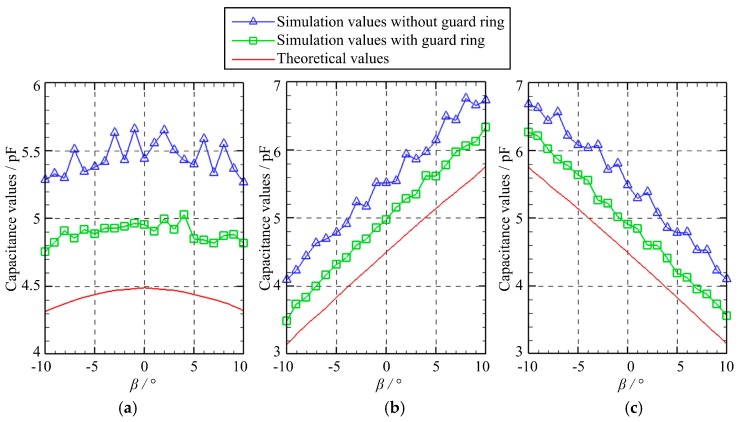
The capacitance variation of three capacitors in the case of A: (**a**) *C*_1_; (**b**) *C*_2_; (**c**) *C*_3_.

Figure 5 presents the capacitance variation of three capacitors as the spherical excitation electrode rotates about the *Y*-axis. Specifically, the rotation angle *γ* equals to zero while the rotation angle *β* varies from −10° to 10° with a step of 1°. Several observations can be made. First, the variation of the simulated capacitance values of the capacitors *C*_1_, *C*_2_, and *C*_3_ exhibits a similar trend with that of the theoretical values. Second, the simulated values are larger than the theoretical counterparts, which can be ascribed to the fringe effect [19,20]. Third, the simulated values can be reduced by using the guard ring. Four, the capacitance values of the capacitors *C*_2_ and *C*_3_ exhibit a linear relation with the rotation angle. As the rotation angle *β* increases, the capacitance value of *C*_2_ rises gradually, while the capacitance value of *C*_3_ reduces.

**Figure 6:**
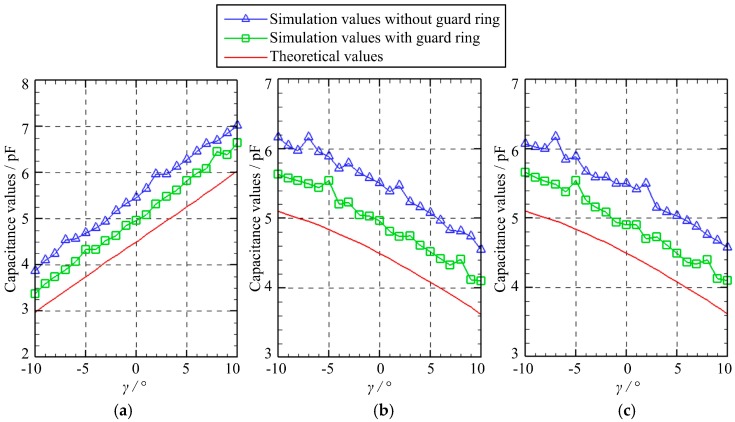
The capacitance variation of three capacitors in the case of B: (**a**) *C*_1_; (**b**) *C*_2_; (**c**) *C*_3_.

Figure 6 presents the capacitance variation of three capacitors as the spherical excitation electrode rotates about the *X*-axis. Specifically, the rotation angle *γ* varies from −10° to 10° with a step of 1°, while the rotation angle *β* equals to zero. Three observations can be made. First, the simulated capacitance values of the capacitors *C*_1_, *C*_2_, and *C*_3_ exhibit a similar variation tendency with the theoretical values. Second, compared with the theoretical values, the simulated ones exhibit an increase of approximately 1 pF, which can be suppressed by using the guard ring. Third, the capacitance values of the capacitors *C*_1_, *C*_2_ and *C*_3_ exhibit a roughly linear relation with the rotation angle. With the rise in the rotation angle *γ*, the capacitance value of *C*_1_ increases gradually, while the capacitance values of *C*_2_ and *C*_3_ decrease.

**Figure 7:**
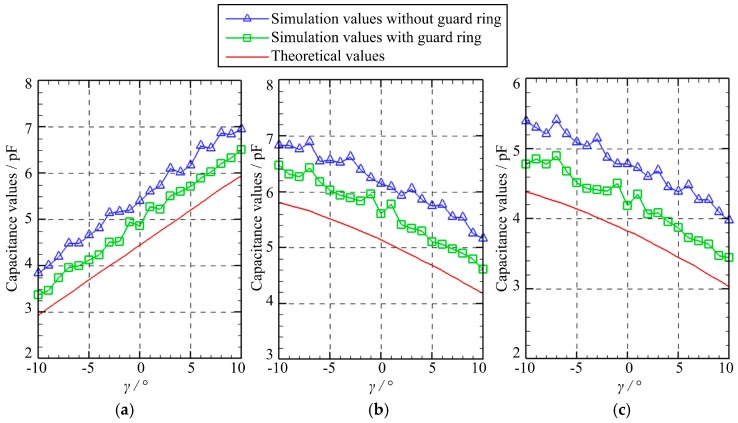
The capacitance variation of three capacitors in the case of C: (**a**) *C*_1_; (**b**) *C*_2_; (**c**) *C*_3_.

Figure 7 presents the capacitance variation of three capacitors as the spherical excitation electrode rotates about the *X*-axis before rotating by a fixed angle of *β =* 5° about the *Y*-axis. The rotation angle *γ* about the *X*-axis varies from −10° to 10° with a step of 1°. Several observations can be made. First, the variation of the simulated capacitance values of the capacitors *C*_1_, *C*_2_, and *C*_3_ is similar to that of the theoretical values. Second, the simulated values with the guard ring are smaller than those without the guard ring, but larger than the theoretical ones. Note that the fringe effect is neglected in the theoretical calculations; thus, it indicates that the guard ring could reduce the fringe effect that occurs in the simulation. Third, as the rotation angle *γ* increases, the capacitance value of *C*_1_ goes up linearly while the capacitance value of *C*_2_ and *C*_3_ drops gradually.

**Figure 8:**
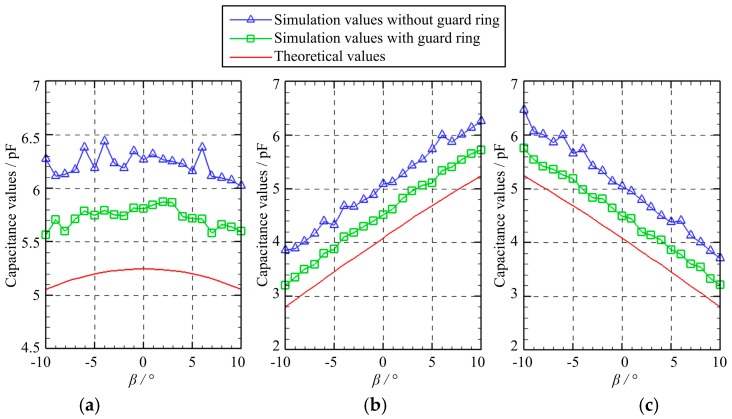
The capacitance variation of three capacitors in the case of D: (**a**) *C*_1_; (**b**) *C*_2_; (**c**) *C*_3_.

Figure 8 presents the capacitance variation of three capacitors as the spherical excitation electrode rotates by a fixed angle of *γ =* 5° about the *X*-axis before rotating about the *Y*-axis. The rotation angle *β* about the *Y*-axis varies from −10° to 10° with a step of 1°. It can be seen that the variation of the simulated capacitance values of the capacitors *C*_1_, *C*_2_, and *C*_3_ shows a similar tendency with that of the theoretical values. However, the theoretical capacitances exhibit smaller values of about 0.5 pF and 1 pF than the simulated counterparts with and without the guard ring, respectively. It should be noted that the capacitance values of the capacitors *C*_2_ and *C*_3_ exhibit a linear relation with the rotation angle about the *Y*-axis. The capacitance of *C*_2_ rises gradually, while the capacitance of *C*_3_ reduces with the increasing angle *β*.

In summary, the variation of the simulated capacitance values of the capacitors exhibits a similar trend with that of the theoretical values. This suggests the feasibility of the proposed method. Note that the proposed sensor consists of three sensing electrodes, which are used for the detection of the angles about the *X*- and *Y*-axes. One may wonder the reason for the adoption of three instead of two or four sensing electrodes. Some reasons are proposed as follows. If two sensing electrodes are placed in the *X*- and *Y*-axes, they could detect the rotational angles about the *Y*-axis and *X*-axis, respectively. However, the angular motion of the spherical joints in practical applications is very flexible. If the rotation axis is perpendicular to the *X–Y* plane, the change in the overlapping area of the spherical plates is relatively small, which would lead to a low sensitivity in the angular motion in the *X–Y* plane. To avoid this drawback, the number of the sensing electrodes should be increased. In this work, three sensing electrodes are distributed axisymmetrically; that is, each electrode is located in the symmetric axis of the other two. As a result, the angular motion in the symmetric plane of each two sensing electrodes can be detected by the third one. Four capacitors could form differential capacitance, which may increase the sensitivity of the sensor. However, there may be the same problem as in the case of two sensing electrodes. In addition, the area of each electrode would be smaller to avoid the structural inference between the sensor and the spherical joint. This may have an effect on the sensitivity of the sensor. Thus, a detailed examination of measurement sensitivity and the structural inference for four sensing electrodes will be made in future works. In fact, we tend to believe that six or more sensing electrodes may be beneficial to achieve the differential capacitance detection. 

## 6. Conclusions

This work proposes a novel method for the rotational angle measurement of a spherical joint based on a spherical capacitive sensor. The 3-DOF motion of a spherical joint is first introduced. Then, the structure of the spherical capacitive sensor is proposed, and the mathematical model of the detection method is deduced. Finally, the spherical capacitive sensor was simulated using Ansoft Maxwell to validate the feasibility of the proposed detection method. The main conclusions that can be made are as follows.

(1) The proposed capacitive sensor consists of a spherical excitation electrode and three spherical sensing electrodes. The excitation electrode is concentrically attached to the moving ball of the spherical joint and the sensing electrodes are concentrically fixed at the inner surface of the socket. Each sensing electrode and the excitation electrode generate a capacitor.
(2) The mathematical model for the rotation angle of a spherical joint and the capacitance of the capacitor is established. The rotation angles (*γ* and *β*) of the output rod of the spherical joint about the *X-* and *Y*-axes could be obtained by measuring the capacitance values of three capacitors. Moreover, the capacitance value of the capacitor has a linear relation with the rotation angle, provided that the rotation angle of the spherical joint is within the range of −10° ~ 10°.
(3) The variation of the simulated capacitance values of the capacitors exhibits a similar trend with that of the theoretical values. This suggests the feasibility of the proposed method. Moreover, the increased capacitance caused by the fringe effect could be suppressed by employing the guard ring and calibrating the sensor.

## Figures and Tables

**Table 1:** Structural parameters of the capacitive sensor.

Parameters	Value
The outer radius of the spherical excitation electrode *r*	24.4 mm
The inner radius of the spherical sensing electrode *R*_0_	25 mm
The outer radius of the guard ring	24.4 mm
The central angle of the sensing electrode *α*_1_	π/6
The central angle of the excitation electrode *α*_2_	π/4
The thickness of the plates *t*	2 mm
The angular clearance between the guard ring and excitation electrode	2°

**Table 2:** Rotation of the excitation electrode (*CE*_d_) in four cases.

Case	Rotational Angle about the *X*-axis (*γ*)	Rotational Angle about the *Y*-axis (*β*)
A	0°	−10° ~ 10°
B	−10° ~ 10°	0°
C	−10° ~ 10°	5°
D	5°	−10° ~ 10°
